# Implementation of an mHealth App to Promote Engagement During HIV Care and Viral Load Suppression in Johannesburg, South Africa (iThemba Life): Pilot Technical Feasibility and Acceptability Study

**DOI:** 10.2196/26033

**Published:** 2022-02-02

**Authors:** Samanta Tresha Lalla-Edward, Nonkululeko Mashabane, Lynsey Stewart-Isherwood, Lesley Scott, Kyle Fyvie, Dana Duncan, Betiel Haile, Kamal Chugh, Yiyong Zhou, Jacob Reimers, Matteus Pan, Maya Venkatraman, Wendy Stevens

**Affiliations:** 1 Ezintsha Faculty of Health Sciences University of the Witwatersrand Parktown South Africa; 2 Molecular Medicine & Haematology University of the Witwatersrand Parktown South Africa; 3 Roche Molecular Systems, Inc Pleasanton, CA United States; 4 Roche Molecular Systems Rotkreuz Switzerland; 5 National Priorities Programme National Health Laboratory Systems Parktown South Africa

**Keywords:** HIV, virological suppression, mHealth, digital health, South Africa, patient-centric, disease management, mobile phone

## Abstract

**Background:**

South Africa has the largest HIV treatment program worldwide. Retention in care and medication adherence remain problematic necessitating innovative solutions for improving HIV care. The increasing availability and use of mobile technology can support positive clinical outcomes for persons living with HIV. iThemba Life is a mobile health app designed with input from South African health professionals and patients, promoting engagement with HIV care through access to medical results.

**Objective:**

This study aimed to test the feasibility and acceptability of receiving HIV viral load (VL) results through the app and compare the time to HIV VL result return for study participants before and after app use.

**Methods:**

Using convenience sampling, adults having routine VL phlebotomy were recruited from 2 Johannesburg health facilities. After signed consent, the app was downloaded on their Android smartphones, phlebotomy was performed, and the sample barcode was scanned through their phone to link the sample and app. Participants received a notification of the result availability and logged into the app to view results, their explanation and recommended action.

**Results:**

Overall, 750 people were screened to enroll 500 participants. Of 750, 113 (15.1%) failed eligibility screening. 21.5% (137/637) had smartphone technical limitations preventing enrollment. Results were released to 92.2% (461/500) of participants’ phones. App technical issues and laboratory operational issues limited the number of released results. Approximately 78.1% (360/461) results were viewed in the app. Median time from notification of availability to result viewed being 15.5 hours (0.6; range 0-150 days). Turnaround time from phlebotomy to the result being received was 6 (range 1-167) days for users versus 56 days (range 10-430 days; *P*<.001) before app use. Overall, 4% (20/500) of participants received unsuppressed results (VL>1000 copies/mL). Turnaround time for unsuppressed results was 7 days for participants versus 37.5 days before app use (*P*<.001). The difference before and after app use in the suppressed and unsuppressed users for time from sample collection to result delivery was statistically significant. Of 20 participants, 12 (60%) returned for a confirmatory VL during the study period. The time from an unsuppressed VL to a confirmatory VL was 106 days for app users versus 203 days before app use (*P*<.001). Overall, 52.4% (262/500) of participants completed an exit survey; 23.2% (58/250) reported challenges in viewing their VL results. Moreover, 58% (35/60) reported that they overcame challenges with technical assistance from others, and 97.3% (255/262) wanted to continue using the app for VL results.

**Conclusions:**

Using iThemba Life for VL results was well-received despite limited smartphone access for some participants. App users received results 10 times sooner than before the app and 5 times sooner if their VL >1000 copies/mL. This increased notification speed led to participants wanting to continue using iThemba Life.

## Introduction

As of 2018, there are 37.9 million people living with HIV (PLWH) worldwide, of which South Africa accounts for 7.5 million (19%) [1]. South Africa has the largest HIV treatment program [2], providing antiretroviral therapy (ART) to 4.4 million people [3]. Despite the high number of treatments, retention of care and medication adherence remain problematic, as only 86% of PLWH successfully achieve viral suppression [4], which is short of the 95% goal outlined by the 95-95-95 Joint United Nations Programme on HIV/AIDS initiative to end the AIDS epidemic by 2030 [5]. Despite increasing viral load (VL) testing, the results do not often lead to changes in the clinical management needed to achieve viral suppression because of clinic attendance and health system factors that delay clinical decision-making [6-8]. Improvements in health care delivery are required to achieve improved clinical outcomes expected with routine VL testing.

The increasing availability and use of mobile technology provide an opportunity for improving health outcomes in low- and middle-income countries [9], and a variety of mobile health (mHealth) interventions have been developed to support the HIV care cascade [10-13]. Traditionally, mHealth platforms have concentrated on text-messaging or phone call interventions [10,11], and SMS which has been shown to improve HIV retention and VL suppression [14]. However, current trends are moving toward app-based interventions that allow the tailoring of content to match the needs and preferences of users and can provide multimedia content to enhance participation and further motivate behavior change. Apps have been shown to be acceptable options in South Africa along the HIV care cascade. In a randomized controlled trial, the SmartLink app was shown to improve linkage to care for those newly diagnosed as HIV-positive by 20% [12]. The Aspect HIVST app has recently undergone a feasibility study in Johannesburg, where the acceptability of the app was high [13].

iThemba Life complements existing conventional ART adherence and viral suppression strategies through the electronic delivery of health information and HIV VL results directly from the laboratory to the user. Our hypothesis is that the iThemba app can provide simplified logistics for timely delivery of HIV VL results and support linkage to care. The objective of this pilot study was to assess the technical feasibility and acceptability of the use of iThemba Life to return HIV VL results and compare the time to HIV VL result return for study participants before and after app use. This is the first report of an evaluation of the iThemba Life app.

## Methods

### Study Design and Participants

In this mixed methods study, 750 HIV-positive adults receiving ART at the Hillbrow Community Health Center and Yeoville Clinic in Johannesburg, South Africa, were screened to enroll a convenience sample of 500 participants. Participants were recruited while waiting to have their blood collected for HIV VL testing and were included if they were aged ≥18 years, comfortable reading English, reported regular access to an Android phone and internet, had blood collected for an HIV VL test on the day of enrollment, and signed the consent form. Participants were excluded if they were not currently on ART, were receiving antenatal care services, had iThemba Life previously downloaded on their phone, or refused to participate. After enrollment, the app was downloaded on their Android smartphones, phlebotomy was performed, and the sample barcode was scanned using their phone camera to link the sample and app.

### Recruitment and Data Collection

A trained, good clinical practice–certified study team, comprising a nurse, recruiter, and counselor, implemented the study at each site. The study team members approached clients, informed them about the study, and invited them to be screened for participation. Potential participants who met the eligibility criteria were provided with an overview of the study, asked to read the informed consent, and then asked to sign it if they agreed to participate.

A study team member assisted with the installation of the app on the participant’s phone (Wi-Fi was provided by study-supported mobile hot spots) and then assisted the participant with setting up an account and provided an overview of app use. Any user unable to download or register on the app was withdrawn from the study, and the consent form was updated to reflect that no medical information would be collected. The recruiter recorded the number of approached clients and the reason for nonparticipation in a screening log for any patient found to be ineligible. If a participant was taken out of the queue for the recruitment process, they had their routine HIV VL collected by the study nurse, and if, instead, they returned to the blood collection queue, their place was maintained. Recruitment took place in April 2019, and participants were followed up until October 2019, when a study exit interview was conducted.

Several logs were maintained during the recruitment and data collection processes (ie, screening log [mentioned above], enrollment log, and VL result log). At enrollment, data collected included ART regimen, ART initiation date, standard time for receiving an HIV VL result, HIV status disclosure, type of phone, and perceived level of HIV knowledge. The VL result log contained the results received through iThemba Life and previous results extracted from the user’s medical records. In addition, there were in-app user experience questions. All users were invited to participate in an exit interview. The interviews were conducted in English using a structured interview guide. Data were collected on demographics, satisfaction with the app, experiences with viewing results, willingness to recommend the app, likes, dislikes, and recommendations for subsequent iterations. Finally, 2 focus group discussions (FGDs) were conducted with a total of 9 (Yeoville Clinic: n=5, 56%, including n=4, 80% male and n=1, 20% female; Hillbrow Community Health Center: n=4, 44%, who were all female) consenting nurses. The discussions were facilitated in English. Trained staff used a semistructured interview guide to gather information on the nurses’ opinions and perceptions of iThemba Life. FGDs were audio recorded and transcribed verbatim.

Throughout the study, app users received all HIV care from the clinic staff per standard of care and not from the study team. HIV VL testing frequency followed national guidelines, and no additional specimens were collected as part of this evaluation. The samples were transported to a central laboratory for testing following standard sample transport procedures for the health facility. The study team did not provide HIV clinical care to standardize clinical care for app users and nonusers.

### Delivery of Results

**Figure 1:**
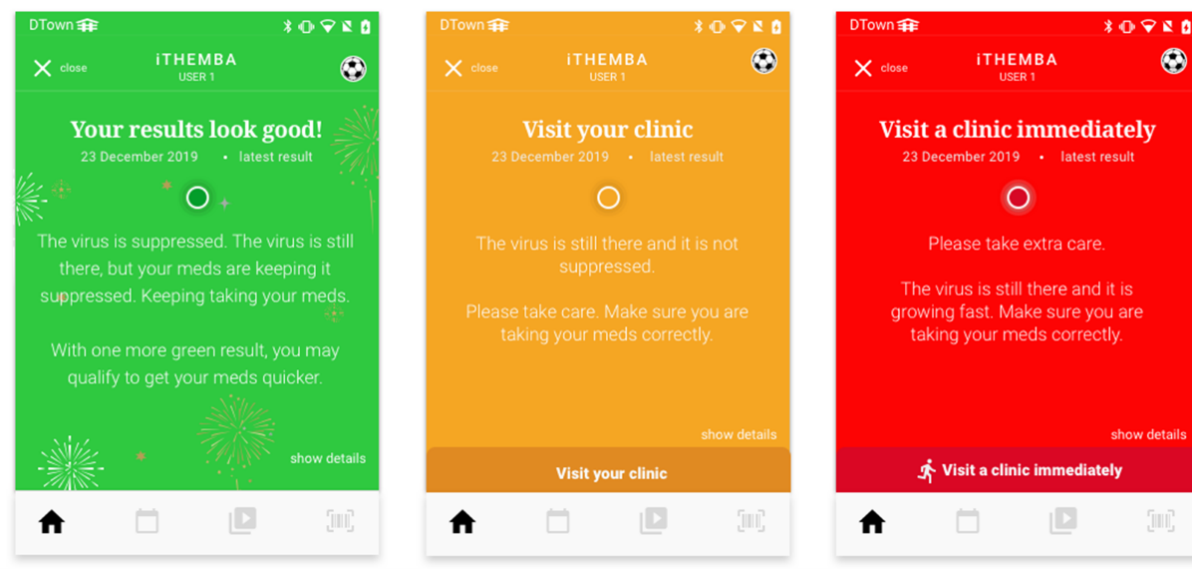
Screenshot of in-app laboratory result explanations.

Once the central laboratory performed the testing, participants received a notification of a message waiting to be viewed on the app. Users could log into iThemba Life to view their results. The laboratory results were presented clearly, with information explaining the results and recommended actions for the user to take (Figure 1). Participants who did not achieve viral suppression were told to return to the clinic for adherence counseling and a repeat HIV VL test in 2 to 3 months according to national guidelines. If participants returned for another HIV VL test during the study period, they scanned their sample barcode with their phone to link the specimen and the app and followed the same procedure described above. Participants who did not view their results were sent a reminder notification 3 days and 7 days after the result was available.

**Figure 2:**
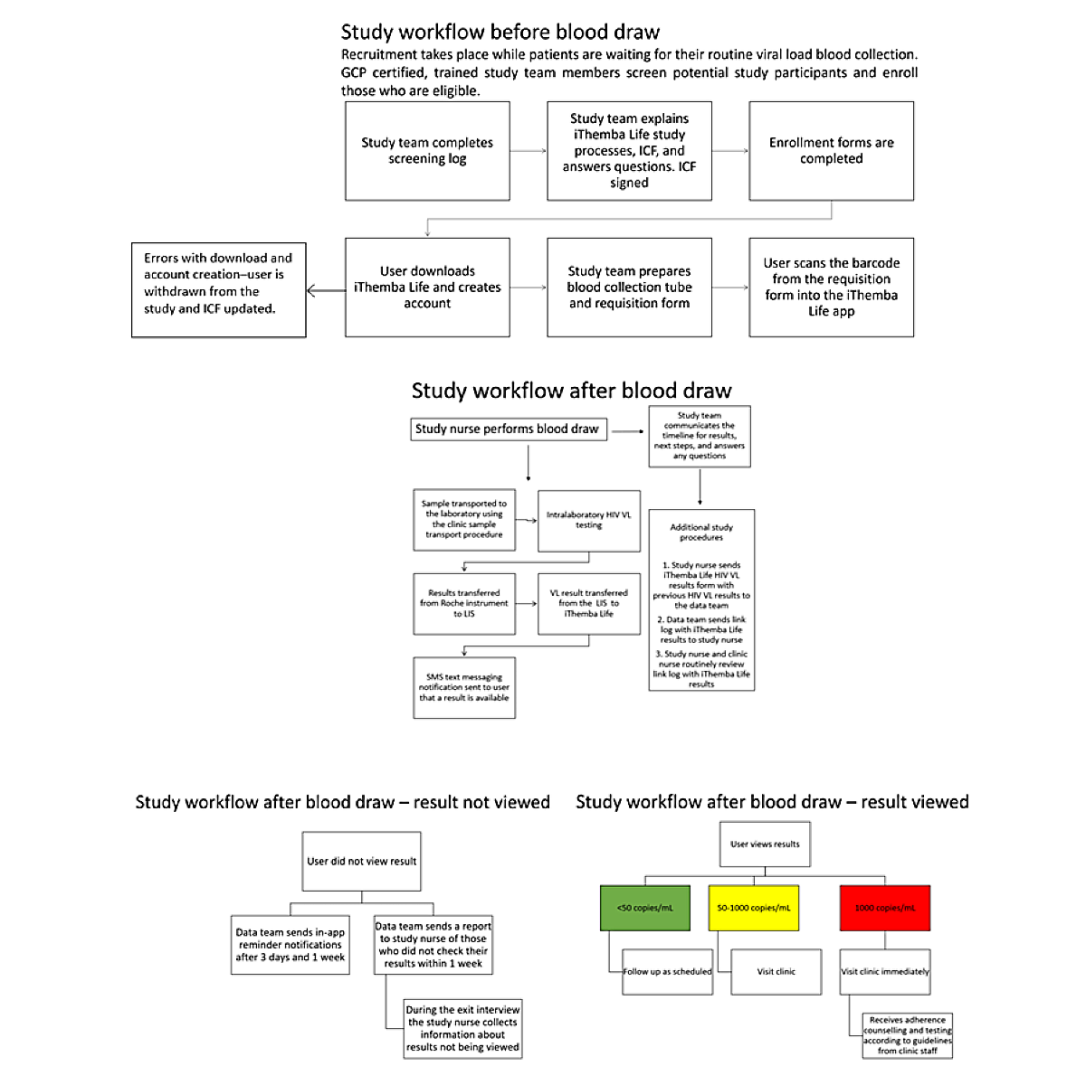
Study workflow. GCP: good clinical practice; ICF: informed consent form; LIS: laboratory information system; VL: viral load; SMS: short messaging service.

The study flow describing the recruitment, data collection, and delivery of results is shown in Figure 2.

### Outcome Measures

The primary objective was to assess the technical feasibility and acceptability of the use of iThemba Life to return HIV VL results. The secondary objective was to compare the time to HIV VL result return for study participants before and after app use.

The technical feasibility of receiving HIV VL results through the app was assessed by process indicators, including the number of individuals screened, HIV VL specimens collected, HIV VL results returned, HIV VL results viewed, time to viewing HIV VL result, and the time to next clinic visit for those with unsuppressed HIV VL requiring follow-up according to national HIV care and treatment guidelines. The acceptability of using the app to return HIV VL results was assessed through in-app user experience surveys using a 5-point Likert scale and through a structured study exit interview. The interviews were conducted in English using a structured interview guide. The collected data included demographics, experience with viewing the results, willingness to recommend the app, likes, dislikes, and recommendations for subsequent iterations. FGDs were conducted with health care workers from the study sites to understand their opinions and perceptions of the app.

To assess the impact of the app on service delivery, study staff extracted data from health facility registers used for routine patient management and from enrollment questionnaires, including the most recent HIV VL results, date of the results being communicated to the individual, and the date of subsequent clinic visits for those with an unsuppressed HIV VL. The median time between sample collection and HIV VL result return was compared for study participants’ before app use when receiving routine service delivery and after app use when receiving app-supported service delivery. As an exploratory end point, the time from the result being received to repeat, confirmatory VL for individuals who were unsuppressed was analyzed. Both the World Health Organization and South African HIV Care and Treatment Guidelines recommend a confirmatory VL before ART regimen change [15,16].

**Figure 3:**
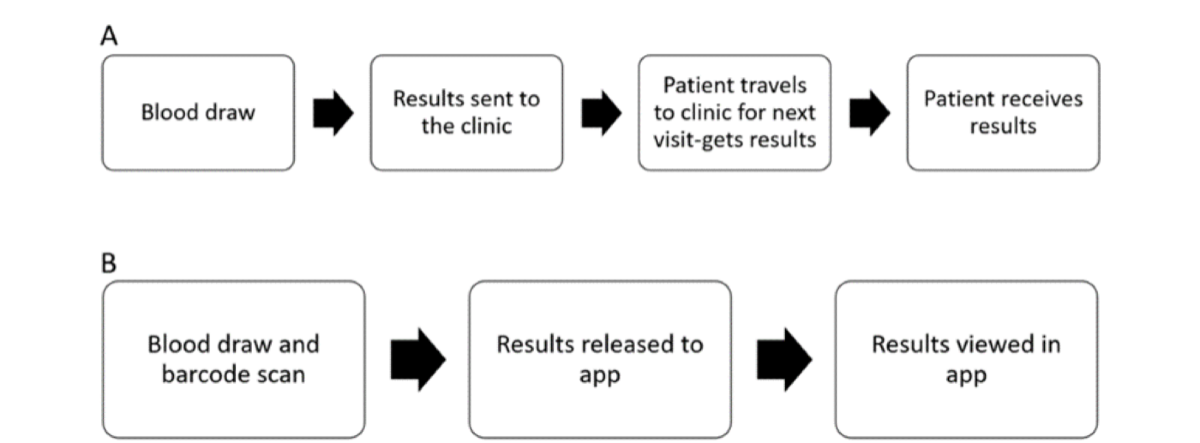
Routine HIV viral load result return (A) before app use and (B) app-supported HIV viral load result return workflow.

The logistics required for HIV VL result return in routine service delivery is complex and often delays result delivery [17]. The use of the app allows for direct communication between the laboratory and the app user, reducing the time from sample collection to the results being returned to the individual and potentially reducing the time to a confirmatory VL and clinical management when needed (Figure 3).

### Data Analysis

Quantitative data were analyzed with descriptive statistics using SAS (version 9.4; SAS Institute Inc). A chi-square test was performed to evaluate the relationship between demographic factors and time to results being viewed after notification of result availability. The Fisher exact test was used to compare the acceptability of the app by age, and a median 2-sample test was used to compare time from sample collection to result delivery before and after app use. *P*<.001 was considered significant. Qualitative data involved thematic analysis using SAS (version 9.4).

### App Development

iThemba Life was developed by Roche Molecular Systems and designed with the input and feedback of >40 health care professionals and >60 PLWH in South Africa. Demonstrations of prototypes provided qualitative insights used to improve and refine the app in preparation for the pilot.

iThemba Life integrates with the local laboratory information system or results database and allows the delivery of any laboratory result from any manufacturer.

The iThemba Life mHealth platform safeguards and manages sensitive patient data. iThemba Life is designed to track sensitive data, both in transit and at rest in the cloud, with prevailing data privacy and security regulations in mind. The security mechanisms of iThemba Life are built on the principles of segregation, minimization, encryption, and monitoring.

Under the principle of minimization of protected health information, users’ names or ID numbers were not collected. The collected data included the phone number, gender, year of birth and name of clinic, and the HIV VL result from the laboratory results database. Data privacy was considered as a design principle throughout the system development. As a result, data collection and representation sought to minimize the risk of disclosure and maximize user privacy. The app icon and name were not suggestive of a health app, and all app-based push notifications provided no detail about the nature of the information, ensuring that all health-related details were only displayed within the password-protected section of the app. Users could delete their profile information from the iThemba Life platform whenever they so chose to comply with local data rights and privacy laws. This would not delete any data that the laboratory or clinic had in their medical files.

### Ethical Considerations

The human research ethics committee of the University of the Witwatersrand provided approval for the study (M181025), and permission was granted by the Department of Health Johannesburg Health District office (National Health Research Database reference GP_201810_009). Trained study staff obtained written informed consent from all the study participants. Participants completing the exit interview received a ZAR 150 (US $10.70) reimbursement for their time.

## Results

### Screening and Enrollment

**Figure 4:**
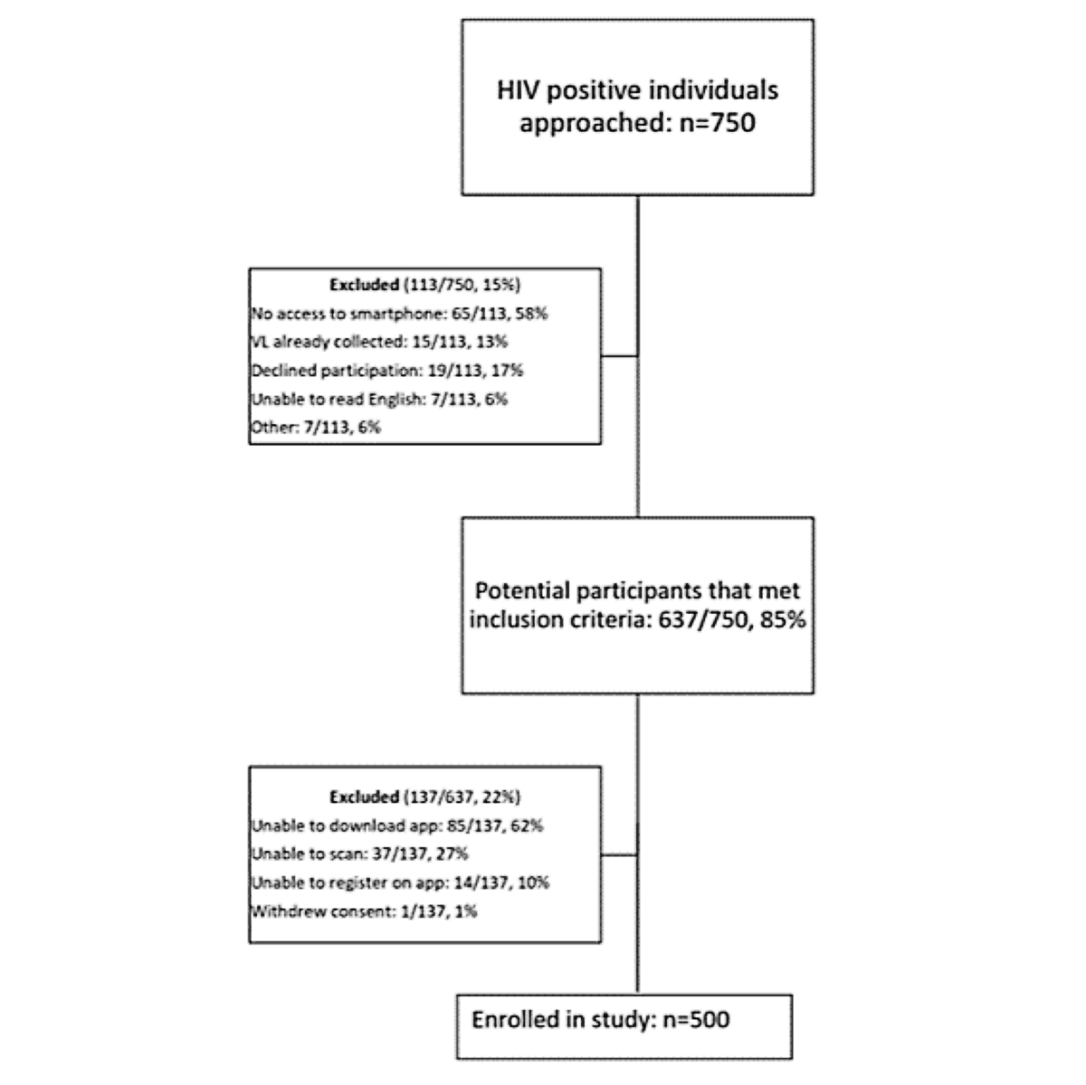
Recruitment flow. VL: viral load.

Over the course of the study, 750 (Hillbrow Community Health Center n=383, 51.1% and Yeoville Clinic n=367, 48.9%) people were screened to enroll 500 (66.7%; Hillbrow Community Health Center n=238, 47.6% and Yeoville Clinic n=262, 52.4%) participants (Figure 4). Of the 750 people, 113 (15.1%) did not meet inclusion criteria, as 65 (57.5%) people did not have access to a smartphone, 15 (13.3%) had a VL sample already collected, 19 (16.8%) declined to participate, 7 (6.2%) could not read English, and 7 (6.2%) were excluded for other reasons. Of the remaining 637 people, 137 (21.5%) of those eligible were not enrolled because of their smartphone’s technical limitations, as 85 (62%) individuals were unable to download the app, 37 (27%) were unable to scan the sample barcode, 14 (10.25%) were unable to register on the app, and 1 (0.7%) withdrew consent.

### Participant Characteristics

Of the 500 participants, 325 (65%) were female. The median age of the patients was 37 years. Of the 500 participants, age disaggregation comprised 3 (0.6%) participants being aged <20 years, 79 (15.8%) being aged 21 to 30 years, 246 (49.2%) being aged 31 to 40 years, 139 (27.8%) being aged 41 to 50 years, 27 (5.4%) being aged 51 to 60 years, and 1 (0.2%) being aged >60 years. Approximately 91.8% (459/500) of users were on first-line ART (tenofovir disoproxil fumarate/lamivudine [or emtricitabine]/efavirenz), whereas 8% (40/500) were on second-line treatment (zidovudine/lamivudine/ritonavir-boosted atazanavir [or ritonavir-boosted lopinavir]), and 0.2% (1/500) had an unknown treatment regimen. Of the 500 participants, 249 (49.8%) could not recall how long it usually took to receive their VL result after a blood draw, and 234 (46.8%) stated that it took >1 month to receive their VL result.

Regarding disclosure of HIV status, although most (481/500, 96.2%) had disclosed their HIV status to someone in their household/family, there was concern about disclosure of their status to others (303/500, 60.6%). Just under half (247/500, 49.4%) of the participants reported that they were very informed about HIV, and some (54/500, 10.8%) participants reported that they regularly used a shared phone. Table 1 shows the baseline characteristics of the study participants.

The exit interview was completed by 52.2% (261/500) of participants: 52.1% (136/261) from Hillbrow Community Health Center and 47.9% (125/261) from Yeoville Clinic. Most (209/261 80.1%) had completed high/secondary school, and some (133/261, 50.9%) were employed full-time. Approximately 70.1% (183/261) of participants earned <ZAR 5000 (US $357) per month. Most respondents (218/261, 83.5%) reported having consistent network coverage throughout the day, and some (197/261, 75.5%) stated that they would have downloaded the iThemba Life app without access to free Wi-Fi at the health facility. Users also reported that the median time to travel to the clinic was 30 minutes, at the cost of <ZAR 20 (US $1.40) for 51.3% (134/261) of participants. A median of 2 hours was spent at the clinic, and 96.2% (251/261) reported that they received the services they required at their last clinic visit.

**Table 1:** Demographic and other characteristics of participants (N=500).

Characteristics		Overall (N=500)	Hillbrow Community Health Center (n=238)	Yeoville Clinic (n=262)
**Sex, n (%)**				
	Male	175 (35)	79 (33.2)	96 (36.6)
	Female	325 (65)	159 (66.8)	166 (63.4)
**Age (years), n (%)**				
	<20	3 (0.6)	2 (0.8)	1 (0.4)
	21-30	79 (15.8)	30 (12.6)	49 (18.7)
	31-40	246 (49.2)	111 (46.6)	135 (51.5)
	41-50	139 (27.8)	75 (31.5)	64 (24.4)
	51-60	27 (5.4)	16 (6.7)	11 (4.2)
	>60	1 (0.2)	1 (0.4)	0 (0)
	Unknown	5 (1)	3 (1.3)	2 (0.8)
**Age (years)**				
	Values, mean (SD; range)	38 (7.8; 19-61)	38 (8; 19-61)	37 (7.5; 19-60)
	Values, median (IQR)	37 (32-42)	39 (33-44)	37 (32-42)
**ARV^a^** **treatment, n (%)**				
	First-line: TDF^b^/3TC^c^ (or FTC^d^)/EFV^e^	459 (91.8)	203 (85.3)	256 (97.7)
	Second-line: AZT^f^/3TC/ATV(r)^g^ or LPV(r)^h^	40 (8)	34 (14.3)	6 (2.3)
	Unknown	1 (0.2)	1 (0.4)	0 (0)
**Have users disclosed status to anyone in household or family?, n (%)**				
	Yes	481 (96.2)	222 (93.3)	259 (98.9)
	No	19 (3.8)	16 (6.7)	3 (1.1)
**User concerned about disclosing condition to others?, n (%)**				
	Very concerned	91 (18.2)	47 (19.7)	44 (16.8)
	Concerned	78 (15.6)	63 (26.5)	15 (5.7)
	Somewhat concerned	134 (26.8)	69 (29)	65 (24.8)
	Not concerned	60 (12)	31 (13)	29 (11.1)
	Not at all Concerned	137 (27.4)	28 (11.8)	109 (41.6)
**Informed about HIV, n (%)**				
	Very informed	247 (49.9)	119 (50)	128 (48.9)
	Somewhat informed	231 (46.2)	112 (47.1)	119 (45.4)
	Not informed	22 (4.4)	7 (2.9)	15 (5.7)
**Shared phone, n (%)**				
	Yes	54 (10.8)	23 (9.7)	31 (11.8)
	No	446 (89.2)	215 (90.3)	231 (88.2)

^a^ARV: antiretroviral.

^b^TDF: tenofovir disoproxil fumarate.

^c^3TC: lamivudine.

^d^FTC: emtricitabine.

^e^EFV: efavirenz.

^f^AZT: zidovudine.

^g^ATV(r): atazanavir/ritonavir.

^h^LPV(r): lopinavir/ritonavir.

### Technical Feasibility

All participants were able to download and register for the iThemba Life app and successfully scan the sample barcode (Table 2). Of the 500 sample bar codes scanned, 461 (92.2%) HIV VL results were obtained from the app database, and of these 461 results, 360 (78.1%) were viewed by users.

All participants managed to scan in their barcoded samples; however, only 92.2% (461/500) results were linked to the iThemba Life database. Of those, most of the results (360/461, 78.1%) were viewed by the participants.

**Table 2:** Feasibility of receiving viral load results through the app (N=500).

	Overall (N=500)	Hillbrow Community Health Center (n=238)	Yeoville Clinic (n=262)
Successful initial viral load samples scanned	500 (100)	238 (100)	262 (100)
Viral load results received by app database	461 (92.2)	218 (91.6)	243 (92.7)
Viral load viewed by user	360 (78.1)	178 (81.7)	182 (74.9)

### Acceptability

Of the 500 users, 480 (96%) responded on the ease of scanning barcode questions through in-app surveys, and of these 480 users, 347 (72.3%) found it easy to perform. When asked about the ease of logging into the app, 52.2% (261/500) of users responded, and of these, 78.2% (204/261) found it easy to perform. For the question on the ease of receiving a VL result through iThemba Life, 19.2% (96/500) of users responded, and of these, 82.3% (79/96) found it easy.

During the exit survey, 79.1% (204/258) of respondents reported that they were very happy with the app, 79.6% (191/240) completely trusted the information from the app, 75.7% (196/259) were likely to recommend the app to others, with 78.4% (203/259) reporting that they already told someone else about the app.

Most participants (227/244, 93%) reported that the app was very helpful, 85.5% (207/242) reported it was easy or very easy to understand the result, and 97.3% (255/262) reported that they wanted to continue to use the app to receive their HIV VL results. Most participants noted the benefit of the app in assisting with the self-management of their disease. Some of their statements included the following:

Most of the time when I get results no one explains them to me but with iThemba Life I managed to get an explanation.

Yeoville Clinic, female

It helped me because I didn’t have to spend money to come to fetch my results

Yeoville Clinic, male

Because it was easy to open it and get results immediately

Yeoville Clinic, female

In the exit survey, of the 500 people, 55 (11%) noted experiencing challenges while viewing their VL results. These included opening the result (30/55, 55%) and data and network access (16/55, 27%) issues. Considering the challenges experienced with iThemba Life (60/500, 12%), these were primarily because of limited technical skills, and many (35/60, 58%) reported that they overcame the challenge with technical assistance from others. Challenges that could not be resolved were because of factors beyond the user’s control (eg, lost phone or broken phone). Of the 500 participants, <3% (n=7, 1.4%; n=4, 57% from Hill Brow Community Health Center and n=3, 43% from Yeoville Clinic) indicated that they did not want to continue using the app. Similar to the challenges reported above, these were not for app-related reasons but rather for other reasons such as no longer having a phone.

The age distribution of the participants who responded to the acceptability questions is reported in Multimedia Appendix 1. There was no statistically significant difference in the acceptability of the app for any age category.

When asked about potential future features of the app, >248 respondents wanted health information and reminders for clinic appointments and medication pickups sent by the app, whereas 75.1% (196/261) participants wanted a daily reminder to take their medication. Some of their requests included the following:

Give information on types of food we can eat or not.

Hillbrow Community Health Center, female

To get updates about HIV every month.

Hillbrow Community Health Center, female

During the FGDs, nurses noted the value of iThemba Life for the users and for themselves. Some of their statements included the following:

I have seen patients showing the result with excitement. Before iThemba Life, they never discussed their viral load.

Hillbrow Community Health Center, nurse

It will be useful, instead of sending the patient to obtain their result they will have access to it.

Hillbrow Community Health Center, nurse

It cuts the time when I need to contact the patient. There will be time saved.

Yeoville Clinic, nurse

Two patients came with high viral load and they were referred to the clinic and switched their treatment.

Hillbrow Community Health Center, nurse

### Service Delivery Outcome

Of the 360 results viewed by app users, 245 (68.1%) were <50 copies/mL, 92 (25.6%) were 50 to 1000 copies/mL, and 20 (5.6%) were >1000 copies/mL. Approximately 0.6% (2/360) of results were reported as invalid and 0.3% (1/360) that was reported as an inadequate specimen by the laboratory.

The overall median number of days from sample collection to the result being received for the app users was 6 (range 1-167) days, whereas the median number of days before app use was 56 days (range 10-430 days; *P*<.001; Table 3). The median time from notification of result availability to results being viewed was 0.6 (range 0-150) days. For unsuppressed users, the overall median days from sample collection to results being received was 7 days for app users and 37.5 days before app use (*P*<.001). For suppressed users, the overall median days from sample collection to the results being received was 6 days for app users and 56 days before app use (*P*<.001). An exploratory analysis of the time to a confirmatory HIV VL found that 60% (12/20) of app users with unsuppressed results returned for a confirmatory VL within the study period. The time from an unsuppressed VL to a confirmatory VL was 106 days for app users and 203 days before app use (*P*<.001).

Before app use, participants would have a VL sample collected and receive their results at their next scheduled appointment, which could be 1 to 6 months later. When there were unsuppressed results, the facility may call the patient to return to the facility sooner. This telephonic follow-up is not consistent and contingent on the availability of both human resources and a working phone and whether the results were reviewed by clinic staff when the facility received them from the laboratory.

**Table 3:** Time from sample collection to result delivery before and after app use (N=500)^a^.

Results delivery		Overall, median	Participants, n (%)	P value^b^
**Number of days from specimen collection to result receipt: all users (days)**				
	Before app use^c^	56.0	251 (50.2)	<.001
	App use	6.0	360 (72)	—^d^
**Time from notification of result availability to results being viewed (hours)**				
	App use	15.5	360 (72)	—
	Before app use	—	—	—
**Number of days from specimen collection to result receipt: unsuppressed users (days)**				
	Before app use^e^	37.5	14 (2.8)	<.001
	App use	7.0	20 (4)	—
**Number of days from specimen collection to result receipt: suppressed users (days)**				
	Before app use	56.0	234 (46.8)	<.001
	App use	6.0	337 (67.4)	—
**Number of days from first to second sample collection: unsuppressed users (days)**				
	Before app use	203.0	27 (5.4)	<.001
	App use	106.0	12 (2.4)	—

^a^Users included in this table have either preapp or app use turnaround data.

^b^Median 2-sample test was used to estimate the P values.

^c^The standard-of-care viral load result delivery times for nonapp users were determined by the date of the next visit after viral load sample collection for users before using the app.

^d^Not available.

^e^The standard-of-care viral load result delivery times for unsuppressed nonapp users were determined by the result delivery times for viral load results before using the app users.

### Association Analysis

The chi-square tests of independence were performed to examine the relationships between the results viewed and age, sex, and time since ART initiation and results were not significant (Table 4).

**Table 4:** Sociodemographic characteristics and feasibility of receiving HIV viral load (VL) result through a mobile app (N=500)^a^.

Variable		HIV VL result received through mobile app	HIV VL result received through mobile app (viewed)	HIV VL result received through mobile app (not viewed)	P value^b^	
**Age (years), n (%)**						.12
	18-25	26 (5.2)	21 (4.2)	5 (1)		
	26-45	359 (71.8)	287 (57.4)	72 (14.4)		
	46-65	71 (14.2)	49 (9.8)	22 (4.4)		
	Unknown	5 (1)	3 (0.6)	2 (0.4)		
**Sex, n (%)**						.25
	Male	165 (33)	124 (24.8)	41 (8.2)		
	Female	296 (59.2)	236 (47.2)	60 (12)		
	Unknown	0 (0)	0 (0)	0 (0)		
**Time since ART^c^ initiation (years), n (%)**						.64
	<1	31 (6.2)	9 (1.8)	22 (4.4)		
	1-2	103 (20.6)	22 (4.4)	81 (16.2)		
	3-5	147 (29.4)	29 (5.8)	118 (23.6)		
	>5	150 (30)	36 (7.2)	114 (22.8)		
	Unknown	30 (6)	25 (5)	5 (1)		

^a^Only participants who received HIV viral load through the mobile app are included in this table.

^b^Chi-square test of independence was used to estimate P values. Data from unknown categories were not included in the P value estimates.

^c^ART: antiretroviral therapy.

## Discussion

### Principal Findings

Similar to a few other digital health efforts aimed at supporting HIV care [12,13,18], this intervention demonstrated notable outcomes and was highly accepted by users. Although the SmartLink adherence app increased linkage to care in youth by 20% [12], iThemba Life users received VL results 10 times sooner than before app use (6 days vs 56 days) and 5 times sooner (7 days vs 37.5 days) than before app use if their HIV VL was >1000 copies/mL. There was a significant difference before and after app use in both suppressed and unsuppressed users with respect to time from sample collection to result delivery. Faster delivery of HIV VL results may significantly improve clinical outcomes.

As HIV/AIDS programs have matured, the fast, reliable delivery of laboratory results has become essential to achieve disease elimination. Digital health can be a powerful tool for increasing the delivery of, and access to, high-quality health services [19]. Front-line health workers are often considered as first-level recipients of digital health [19]; however, the increasing availability of smartphones facilitates increased patient engagement by promoting a shift from text-based to interactive app-based solutions [20].

Access to smartphone technology is rapidly increasing, leapfrogging traditional service delivery constraints, and overcoming infrastructure barriers. The penetration of smartphones is not yet universal throughout Sub-Saharan Africa but has accelerated significantly in recent years with the availability of lower-priced devices, and it is expected to continue increasing to 67% in 2025 [21]. iThemba Life leverages the increasing availability and use of mobile technology to simplify the manual and complex logistics currently used for HIV VL result delivery and ensures that results are available for timely clinical decision-making. The app was designed to complement existing conventional ART adherence and viral suppression strategies through the electronic delivery of health information and HIV VL results directly from the laboratory to the user. This is the first report of an evaluation of the iThemba Life app.

iThemba Life followed an iterative development strategy with continual input and collaboration from patients, health care workers, and other key stakeholders to address sociocultural and workflow aspects related to HIV program delivery. During the development of iThemba Life, focusing on understanding user needs and experience with early prototypes, limiting the installation size, and ensuring that the app could be used on older and less expensive smartphones was essential. This early focus ensured that operational and technical challenges experienced by similar interventions in this setting [22] were minimized, and most of those screened in this study were able to access the intervention and had a positive experience with the app.

Data costs are considered high by many South Africans and typically hinder the uptake of digital health interventions [23]; however, most exit survey respondents reported that they would still have downloaded the app without free Wi-Fi access during this pilot. This not only qualifies satisfaction with the app but also highlights health care recipients’ urgent need for improved service delivery in that costs were outweighed by the ability to receive HIV VL results sooner. iThemba Life users recognized the value of quickly receiving their VL result as a motivator of adherence to treatment, a way to make health management more convenient, and as a source of health information. Many felt that the app was supportive in managing their disease and equipping them to assume control of their health. Health care workers echoed this value of the app for users and noted the benefits of having more informed clients and reliable access to VL results.

Innovative app-based technologies are accompanied by challenges concerning the phone capability and technical proficiency of users, and these areas have been prioritized for improvement following this pilot assessment. The inability to use this app on certain phones or by some users created barriers to entry for a few individuals and has been addressed, as iThemba Life is positioned to scale up with updates to the software to decrease technical challenges.

However, despite these challenges and recent studies advocating for non–app-based mHealth interventions [18,24], the high level of user acceptability coupled with the marked decrease in VL result delivery times indicates that iThemba Life provides value to potential users and the national HIV program. iThemba Life users with unsuppressed results returned for a confirmatory VL in half the typical time they would have, which may decrease the time spent on an ineffective ART regimen, potentially having individual health and population benefits by lessening the time to viral resuppression. The ability to receive VL results electronically decreases visits to health care facilities, which may reduce strain on facilities while also saving patients’ time and money. South Africa has a proven track record of promoting and adopting innovative mHealth interventions such as MomConnect [25], which has >2 million registered users who receive prenatal support messages through the platform [26]. If South Africa was able to similarly scale up iThemba Life, PLWH could have significant financial and time savings on transportation and clinic waiting [27] while also improving their health outcomes and reducing the risk of transmission because of earlier resuppression times.

Using smartphones, iThemba Life is at the beginning of a new frontier of communication between patients and health care workers. This evolving platform will continually incorporate feedback from active users to improve its functionality and features. In the future, iThemba Life can continue to deliver information and services to patients and also create bidirectional feedback from patients to further assess the quality of health care delivery and foster a richer, more meaningful interaction with the health system. In addition, through smartphone technologies, iThemba Life offers the capability to create password-protected multiple user profiles on a single device to allow a device to be shared by families or communities, further expanding access to these services.

### Limitations

Because of the mobile requirements of the app, connectivity issues and incompatibility with some phone models prevented some prospective users from participating. Furthermore, some of the phone cameras were not able to scan the barcode because of the fixed focal length of the camera, which did not permit close-up scanning of the barcode. A dry run of the complete app workflow was not conducted before the start of the study. Consequently, some app technical issues were not identified until after the study enrollment began. Technical issues with the app database itself and operational issues because of sample transport and testing limited the number of results released to the app. The placement of the user survey within the app flow resulted in many users not viewing and, consequently, not completing some or all of these in-app questions. The study staff faced challenges with users returning for the exit survey, which was due in part to the 2019 Johannesburg riots, limiting travel to the sites.

Johannesburg is a large urban center with more resources and services than many other cities in the country and on the African continent; therefore, the current data are not generalizable outside of Johannesburg. In addition, most participants in this study had been receiving ART for many years and were virally suppressed. Future studies would need to target users who have recently initiated ART or who are facing challenges in achieving or maintaining viral suppression.

### Conclusions

This evaluation demonstrated the feasibility and acceptability of receiving HIV VL results directly to users’ smartphones through the iThemba Life app while also decreasing the time for PLWH to receive their VL results. iThemba Life has the potential to increase treatment adherence and literacy, reduce barriers to entry for special populations, and reduce the burden of care for PLWH. Future studies are needed to further our understanding of how the support and information provided by iThemba Life affect the health outcomes of PLWH and how iThemba Life can support other disease areas.

## Appendix

Multimedia Appendix 1 Acceptability of iThemba app by age (years; N=500).

## Abbreviations

ART: antiretroviral therapy
FGD: focus group discussion
mHealth: mobile health
PLWH: people living with HIV
VL: viral load
